# Gunshot Abdominal Injuries: A Report of Two Cases and a Review of the Literature

**DOI:** 10.3390/medicina59101713

**Published:** 2023-09-25

**Authors:** Zlatan Elek, Gojko Igrutinovic, Blagoje Grujic, Ivona Djordjevic, Strahinja Konstantinovic

**Affiliations:** 1 Clinical Hospital Center, 38220 Kosovska Mitrovica, Serbia; 2 Faculty of Medicine, University of Pristina, 38220 Kosovska Mitrovica, Serbia; 3 Institute for Mother and Child Health Care “Dr Vukan Čupić”, 11070 Belgrade, Serbia; 4 Faculty of Medicine, University of Belgrade, 11000 Begrade, Serbia; 5 Clinic for Pediatric Surgery, Orthopedics and Traumatology, University Clinical Center, 18000 Nis, Serbia; 6 Faculty of Medicine, University of Nis, 18000 Niš, Serbia

**Keywords:** gunshot, injury, abdominal organs, complication, children, mortality

## Abstract

Abdominal injuries in children caused by guns are a rare clinical entity globally. But, in countries with undefined legal regulations and in war zones, urban violence is a tremendous social problem among older children and adolescents. This manuscript provides details regarding two cases of severe gunshot injuries in young children. The injuries were very complicated and included damage to the parenchymatous and hollow organs and major blood vessels. The clinical presentation on admission was severe and dramatic, but the patients survived. However, one patient developed numerous complications that required repeated surgical interventions and long treatment. This article provides a detailed description of injuries and how to treat them. Patient care requires a multidisciplinary approach, and the initial decision on further treatment depends on the patient’s hemodynamic stability.

## 1. Introduction

Abdominal injuries in children caused by guns are a rare clinical entity globally. Gunshot-related injuries lead to over 20,000 emergency department visits in children per year in the USA as a leading cause of death [1]. Recent data from the Centers for Disease Control and Prevention report increases in the death rates by firearm in pediatric patients up to 19 years in the past two decades [2], with a four times higher mortality rate in a black population. Unfortunately, the increasing mortality rate is higher every year, at 0.55 per 100,000 in the last 10 years [3]. During the pandemic, the COVID-19 gunshot-related violence rate was stable because the youth were under the supervision of a parent or guardian [4]. But, in countries with undefined legal regulations and in war zones, urban violence is a tremendous social problem among older children and adolescents [5]. Abdominal injuries can occur as a result of blunt or penetrating trauma. In children, blunt force injuries are much more likely, occurring in approximately 85% of children [6].

Gunshot wounds or different kinds of daggers are the most common sources of penetrating injuries. Data from the literature indicate that guns account for more than 90% of injuries from gunshots in older children (those over 12 years old) and adolescents [7]. According to localization, the most commonly affected sites are the gastrointestinal tract, liver, major blood vessels, kidneys, and spleen [8,9]. All deaths caused by guns are tragic, but those involving young children and adolescents are particularly troubling for the whole family and associated with a large number of potentially fatal complications. This paper reports two cases of young child victims with severe injuries caused by guns, focusing on a review of the literature and the treatment of these patients in conditions of reduced resources.

## 2. Methods

The electronic search for this review included the two databases EMBASE and Google Scholar, and used search terms: “gunshot”, “injury”, “abdominal organs”, “complication”, “children” and “mortality”. The inclusion criteria were articles for which the full text was available and studies conducted in adults and children.

The exclusion criteria were articles that were not in English or were grey literature. From the articles retrieved in the first round of search, additional references were identified by a manual search among the cited references. The search was limited to papers published between 2000 and 2023, and forty-four papers that were confirmed to be eligible for the study were found after the search. Due to a lack of available literature, papers published prior to 2000 were included for some issues.

**Figure 1:**
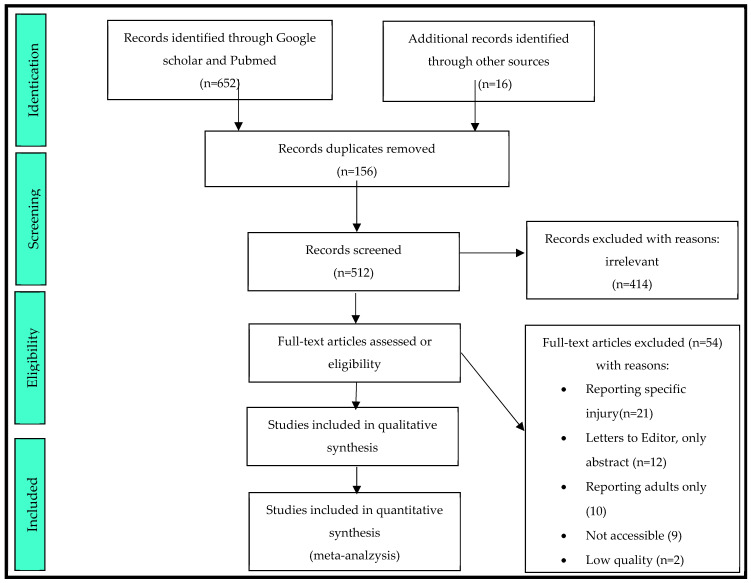
A systematic PRISMA flow chart for the article identification.

A systematic PRISMA flow chart for the identification of articles is presented in Figure 1.

## 3. Results

After reference tracking from a total of 668, only 44 articles were used for the analysis and summary, as is presented in PRISMA flow chart. Of the 44 studies, 23 (52.27%) processed data exclusively related to the pediatric population, while the other 21 (47.73%) studies analyzed the entire population. The majority of studies (19 (43.18%)) dealt with all organ or abdominal organ injuries, 18 (40.90%) dealt with those resulting from gunshots, while a minority (2 (4.54%)) were focused only on one organ (4.54%) (liver, spleen) or two regions (bone fractures and abdominal injuries or abdomen and chest injuries) (2.27%). The sample size varied from 46 to 90,025 patients in the study.

Table 1 summarizes the characteristics of the included studies.

## 4. Case Reports

### 4.1. Case Report 1

A 16-year-old patient was transported by ambulance because of an abdominal injury caused by a firearm shot from a gun. The patient was conscious, oriented, afebrile, tachycardic, and hypotensive with a tension of 80/40 mmHg. The skin was extremely pale and discolored. Physical examination recorded gunshot wounds; the entry wound was located in the right para-umbilical and the exit wound was in the left sacral region.

The patient was clinically, biochemically, and ultrasound-examined and immediately transferred to the intensive care unit. Laboratory values on admission were red blood cells 2.1, leucocytes 15.6, hemoglobin 54, hematocrit 19, and platelets 89. Ultrasound findings (FAST) revealed the presence of free fluid in the abdomen without lesions of solid organs. After emergency resuscitation and blood transfusion, urgent surgical intervention was performed. The patient’s condition was very poor, and additional diagnostic procedures would have contributed to the faster deterioration of the patient.

**Figure 2:**
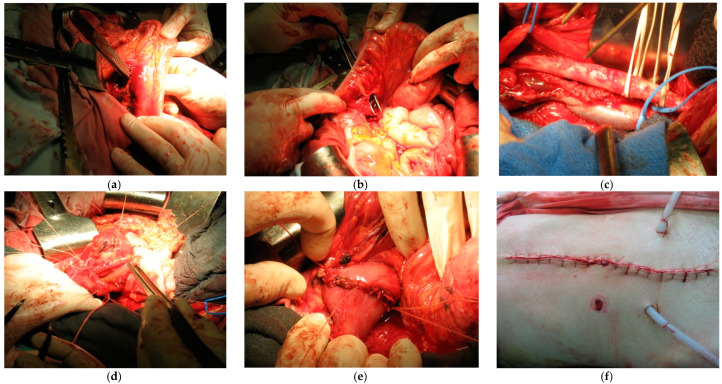
(**a**,**b**) Multiple injuries of the mesocolon transversum and mesentery of the small intestine, (**c**) rupture of D3 duodenal portion, (**d**) laceration of inferior caval vein and right v. iliaca communis. (**e**) The primary suture of the duodenum; (**f**) two drains were placed: the first one in the right paracolic, and the second one in the recto-vesical space.

During the operation, multiple injuries to the mesentery of the transversal colon (Figure 2a,b), the mesentery of the small intestine, laceration of the D3 duodenum, and retroperitoneal hematoma at the level of the femoral fossa on the right were recorded. After the opening of the retroperitoneum, injuries to the inferior vena cava in the form of a 2 cm long laceration and a 3.5 cm long laceration of the right common iliac vein were recorded (Figure 2c). Primary suture of the injured blood vessels was performed. The duodenum was then mobilized by Koher’s maneuver to find the laceration of the duodenum in the D3 area below the ampulla of water (Figure 2d), and the primary suture of the duodenum was performed (Figure 2e). Injuries of the mesentery of the transversal colon and the small intestine were treated with single sutures. Two drains were placed: the first one in the right paracolic and the second one in the recto-vesical space (Figure 2f). The patient spent 5 days in the intensive care unit, treated with triple antibiotic therapy, analgesics, and proton pump inhibitors. On the sixth and seventh postoperative days, the drains were removed. The passage of the gastrointestinal system was established on the third postoperative day, and on the tenth day after the intervention, he was discharged from the hospital. During the two controls, using color Doppler, blood vessels were normal. A month after the surgical intervention, a gastroscopy was performed, and the examination was normal.

### 4.2. Case Report 2

**Figure 3:**
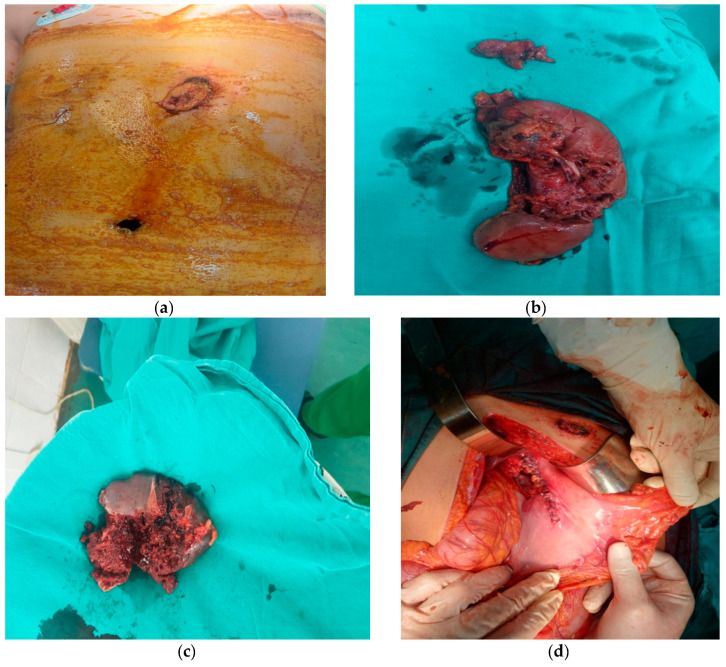
(**a**) Patient with an entry wound in the epigastric area left of the xiphoid, and an exit wound in the left paravertebral area, (**b**) conquassation of the spleen with transsection of the splenic vein and artery, (**c**) lesion of the left kidney with transection of the renal vein and artery, (**d**) primary stomach suture.

A 12-year-old patient was admitted to the hospital with a penetrating abdominal injury inflicted by an automatic rifle. The patient was confused, with no verbal communication, extremely pale, and drenched in a cold sweat. A physical examination recorded an entry wound in the epigastric area left of the xiphoid and an exit wound in the left paravertebral area (Figure 3a).

The patient was transferred to the intensive care unit, and blood was taken for laboratory analysis and blood tests. The patient was extremely hypotensive with a blood pressure of 70/30 mm Hg, and a surgeon decided to perform an urgent surgical intervention only after the transfusion of 2 units of blood. After opening the abdomen, the following injuries were recorded: injury of the anterior and posterior walls of the stomach in the length of 6 cm on the front wall and about 15 cm on the back wall; a complete transection of the pancreas and conquassation of two-thirds of the pancreas in the body and tail area; conquassation of the spleen with transsection of the splenic vein and artery (Figure 3b); lesion of the left kidney with transection of the renal vein and artery (Figure 3c); transsection of the left ureter; lesion of the left lobe of the liver; and complete transection of the left m.quadratus lumborum. About 1800 mL of blood was evacuated from the abdominal cavity. Due to complete devascularization, a splenectomy was performed, followed by a nephrectomy with ureterectomy, a partial pancreatectomy of the distal part of the pancreas, a suture of the head of the pancreas, and a stomach suture (Figure 3d). Drains were placed in the retroperitoneal, left paracolic, and rectovesical spaces. During the surgical intervention, the patient received six units of blood. After the surgery, he was transferred to the intensive care unit. His condition was stable until the nineteenth postoperative day when he deteriorated rapidly due to a massive abdominal hemorrhage due to hemorrhagic pancreatitis, located at the site of the confluence of the left renal artery in the aorta. During the second operation, three drains were placed in the abdominal cavity, as well as intestinal adhesiolysis. Twenty-four days after the surgery, intestinal contents appeared in the drain, and another operation followed. The anastomotic leak was on the anterior wall of the stomach. Lavage of the abdominal cavity was performed with gastrostomy and gastroplasty by Mikulic and with the placement of an open abdomen system (ABTHERA). Twenty-five days after the last intervention, intestinal contents were again in the drain, and reoperation was performed. The colonic gangrene in the area of the hepatic flexure was found, with subsequent right hemicolectomy and Brooke ileostomy, and the open ABTHERA system was again installed. On the twenty-eighth postoperative day after this intervention, the bile content was verified in the drains, which indicated the presence of an intestinal fistula. A new surgical procedure was performed, the ABTHERA system was removed, a protective Stamm–Kaden gastrostomy was performed, drains were replaced, the abdomen was closed with tension, and in the upper part of the wound, a vacuum-assisted wound closure system (VAC) was placed. Finally, after 2 weeks, VAC was extracted, and a contrast passage of the gastroduodenal was performed, as well as a fiberoptic panendoscopy. The patient was discharged from the hospital for home treatment after 128 days. Six months after discharge, the patient was hospitalized again for ileus due to small intestinal adhesions. Surgery was performed, with partial resection of the ileum and T-T anastomosis due to ileum injury during adhesiolysis. The postoperative days went smoothly. He has been operated on six times. Today, he comes regularly for check-ups; his general condition is good, and his local findings are normal.

## 5. Discussion

Firearm injuries among children are a huge clinical and public health problem that has become a growing concern in recent decades, especially in countries where gun ownership is widespread [10,11,12]. According to data from the literature, in 2020, there were 45,222 gunshot-related deaths in the United States, with the highest prevalence in teens and young adults (15–34 years), and even in younger children [13,14]. In recent decades, the incidence of these injuries has been constantly increasing, and these injuries became one of the leading causes of child mortality in the United States of America in 2020 [15]. Intentional injuries are most common among boys over 12 years old (over 80%), as we reported in our study. DiScala reports similar data in his study analyzing data from the National Pediatric Trauma Registry and the United States National Electronic Injury Surveillance System [16]. It is difficult to correlate statistics in the literature because numerous studies include older children and teenagers rather than the younger population of children, with age cut-offs varying from one study to the next. After all, the vast majority include ages of at least mid-teen years.

Most gunshot-related nonfatal injuries are minor, but some are very severe and have more far-reaching consequences and may result in life-long limitations, especially in the pediatric population, with limited reports to date about long-term sequelae. Although they are rare in children, gun injuries are second in terms of mortality after deaths caused by traffic accidents [17,18]. In line with expectations and according to the literature, the vast majority of affected children are boys older than 12 years [19,20], with a higher rate of unintentional firearm-related injuries in the younger ages, regardless of sex.

The severity of firearm injuries is directly proportional to the distance of the projectile fired. Injuries less than three meters away cause massive tissue damage and are usually fatal [8,11]. Thus, tissues with a higher specific gravity suffer more, mostly bone and muscle tissue and parenchymal organs as it is in our cases. The data in the literature concerning the part of the body that is most often injured vary greatly. According to some literature data, the most commonly injured organs are the head and chest [21], while other studies cite the abdominal organs as the most commonly injured [22].

The biomechanical reaction of the abdomen to traumatic damage is correlated with a unique aspect of anatomical structure and physiological response in children. Severe injuries of the abdominal organs are more common in children than in adults and are caused by a smaller area of the abdominal wall and the possibility of expanding the effect of force on more than one abdominal organ at the same time. The visceral organs of the child are relatively larger, more mobile, and located in a smaller compartment space. The abdominal muscles are thinner and abdominal wall has less adipose tissue, which provides a lower level of support and protection from injury [23,24].

The management of patients with penetrating abdominal injuries requires a multidisciplinary approach. The initial step is the application of the advanced life support algorithm using the ABCDE approach. There is no unique standard of treatment for patients with penetrating abdominal injuries. Previously, the initial approach to treatment was urgent surgical exploration. However, analyzing this approach, some authors claim that it is associated with a higher incidence of complications, a higher percentage of unnecessary explorations, and a longer hospital stay. Conservative treatment measures are increasingly gaining importance in hemodynamically stable patients [25,26,27,28,29]. Renz et al. show the frequency of complications after unnecessary laparotomies is as high as 41.3% [30]. Also, laparoscopy can be used for the diagnosis and, in some cases, the treatment of injuries, reducing the percentage of complications that arise as a result of an open surgical approach [31]. But the subject of the greatest debate in the literature, without clearly defined recommendations, is what to do in patients with severe instability. Should we risk the fatal deterioration of the patient due to additional time for diagnosis or perform a surgical intervention as early as possible? How should we react in the conditions of reduced resources and a lack of CT findings?

Further procedures depend on the patient’s hemodynamic stability. Hemodynamically stable patients allow more time for physical examination, laboratory, and radiological diagnosis. But critically unstable patients with very severe injuries require urgent surgical treatment without further diagnosis, as we had to perform in one injured patient. Some authors point out that the preparation of the operating room should begin immediately after the admission of unstable patients [32]. Lynch et al. believe that urgent laparotomy is indicated in patients with pneumoperitoneum, in patients with evisceration of intraperitoneal contents, and in patients in whom hemodynamic instability is maintained despite intensive resuscitation (transfusion of more than 50% of the total blood volume) [33]. There is a large discrepancy in the literature about the method of intravenous resuscitation of fluids or blood derivatives. While some authors recommend intensive resuscitation, others suggest careful intravenous resuscitation until the control of active bleeding is achieved on the operating table. They believe that with limited fluid replacement, maintaining blood pressure at lower values, and limiting dilutional coagulopathy, a better survival rate is achieved [34].

After opening the abdomen, the primary goal is bleeding control. Depending on the severity of the patient’s general condition and the severity of the hemorrhagic shock, the surgeon may initially perform damage control surgery in order to keep the patient alive [35]. This lifesaving procedure has significantly decreased the morbidity and mortality of critically ill patients. The leading cause of mortality in patients with severe trauma of the abdominal parenchymatous organs is hemorrhagic shock (up to 40%) [36] and the principle of damage control surgery has become an option that surgeons prefer rather than focusing on anatomical restoration in the initial surgery. Definitive repair is usually performed in a few days when the patient stabilizes after hemorrhagic shock. The principles of damage control surgery are more widely used in hemostasis and packing for serious injuries of the parenchymatous organs of the abdomen (liver, spleen, and kidneys). But in our cases, the injuries affected large major blood vessels, and insisting on their anatomical restoration was imperative.

The temperature of the patient’s body, the operating room, and the blood products is very important and must be above 35 °C to prevent a lethal triad consisting of hypothermia (<34 °C), acidosis (pH < 7.2) and coagulopathy (aPTT > 55 s) [37,38]. The treatment of hollow organ injuries depends on the severity of the injury. Injuries of solid organs such as the spleen and liver, with bleeding, can be solved with local hemostatic maneuvers, while in more severe cases, resection and suture are required. Treatment of injuries to the main blood vessels of the abdomen is of priority because lesions of these organs are associated with high mortality [32].

Observing the risk factors for the fatal outcome of such injuries, Tyburski et al. believe that an initial systolic pressure of less than 90 mm Hg and a body temperature of less than 34 °C are the most important risk factors for a fatal outcome [39]. Other authors emphasize the importance of base deficit as one of the predictive factors, not only for injury severity but also for fatal outcomes [40].

A number of complications accompanying gunshot injuries have been reported. Potential complications after gunshot injuries are predominantly determined by the site of injury and damaged organs. Except for bleeding, which must be controlled initially (damage control surgery for restoring the anatomy), most common complications are tissue and organ damage, broken bones, wound infections, partial or full paralysis (in the case of spinal cord injuries), and psychological consequences, especially in young patients.

Patients with chest and abdominal injuries are more likely to be re-hospitalized due to complications [41], as was the case in case N°2. The most common complications are wound infection, wound dehiscence, wound infection, sepsis in the early period, small bowel syndrome, and intestinal adhesions with ileus as late complications [42]. The wound infection rate (32.1%) is slightly higher compared to the overall wound infection rate [43], probably because of open bone fractures, perforating wounds, anemia, and generalized poor immune response [44].

Gun violence is a complex and multifaceted problem that requires multi-disciplinary solutions and prevention programs. Violence prevention is a continuous process that begins in early childhood with programs that help parents raise children who are emotionally healthy and who learn to identify and intervene with problematic people or violent people with mental illnesses and how to behave in risky situations and to warn of potential danger.

## 6. Conclusions

Abdominal injuries caused by firearms in recent decades represent a very important cause of morbidity and mortality in children. The incidence varies and is directly proportional to the degree of violence in society. As expected, the dominant number of such injuries is among boys, the average age of which is over 12 years. Patient care requires a multidisciplinary approach, and the initial decision on further treatment depends on the patient’s hemodynamic stability. While hemodynamically stable patients provide the opportunity for additional diagnostic procedures and conservative treatment, hemodynamically unstable patients require immediate surgical intervention.

## Figures and Tables

**Table 1:** Characteristics of the included studies.

Author	Year	n	Age	Country	Data Time Interval	Affected Organs	Recommendations and Conclusions	Type of Study
Flaherty MR et al. [1]	2020	N/A	0–19 y	USA	2010	Abdomen	Developing prevention programs	Review
Andrews AL et al. [2]	2022	0.55/100,000	0–19 y	USA	2001–2019	Abdomen	Developing prevention programs	Cohort study
Hageman JR et al. [3]	2022	N/A	0–18 y	USA	2019–2020	Abdomen	Education about guns and gun safety	Editorial
Stephanie Liou HY et al. [4]	2021	1586	0–19 y	USA	2019–2020	Abdomen	To define counseling strategies for injury prevention	Population-based study
Beraldo LF et al. [5]	2019	107	2–18 y	Brazil	2016–2018	Abdomen	Adolescents are at-risk groups for firearms injuries	Retrospective
Drexel S et al. [6]	2017	N/A	0–19 y	USA	NS	Abdomen	High-risk injuries that require experienced surgeons	Review
Amick LF et al. [7]	2001	N/A	0–19 y	USA	2001	Abdomen	Recommended algorithm for surgery	Review
Stephanopoulos et al. [8]	2023		Children and adults	USA	NS	All organs	Determining diagnostic algorithm	Review
Djordjevic et al. [9]	2016	46	0–18 y	Serbia	2012–2016	Abdomen	Definition of follow-up protocol	Dissertation
Murray CJ et al. [10]	1997	>20,000	Children and adults	Global study	1990	All organs	Collecting the data for a more informed debate on public health priorities	National registry data
Shrestha R et al. [11]	2022	N/A	Children and adults	India	NS	Abdomen	Summarize the systematic examination of gunshot wounds to assist law enforcement effectively	Review
Naghavi M et al. [12]	2016	Over 100,000	Children and adults	Global study	1990–2016	Abdomen	Understanding global variation in firearm mortality rates could guide prevention policies and interventions	Original research
Gurrey S et al. [13]	2021	Over 100,000	Children and adults	USA	2001–2019	Abdomen	Identifying and describing the limitations of firearm injury and prevention programs	Scoping review
Beardslee J et al. [14]	2019	503	0–19 y	USA	2019	All organs	Pediatricians and their parents are in a pivotal position to prevent gun violence	Cohort study
Roberts B et al. [15]	2003	8527	0–19 y	USA	2005/2017	All organs	To create public health strategies across varying vulnerable pediatric populations	Retrospective study
DiScala C et al. [16]	2004	774	0–19 y	USA	2004	All organs	Public policies should be developed and implemented to reduce gunshot injuries	Retrospective
DiScala C et al. [17]	2000	30,708	0–19 y	USA	1998	All organs	There is still a high percentage of deaths after gunshot injuries	Comparative study
Annest JL et al. [18]	1995	99,025	Children and adults	USA	1992–1993	All organs	To provide uniform data on firearm-related injury morbidity and mortality for use in research and prevention efforts	Retrospective comparative study
Saunders NR et al. [19]	2021	5486	0–24 y	Canada	2003–2018	All organs	Firearm injuries are a preventable public health problem. Focus should be on prevention efforts.	Cross-sectional population-based study
Gotsch KE et al. [20]	2001	11,502	Children and adults	USA	1993–1998	All organs	To design and implement prevention programs	Observational study
Nasrullah M et al. [21]	2009	286	Children and adults	Pakistan	2002–2007	All organs	The abdomen and pelvis are the most affected regions	Retrospective study
Lynch T et al. [22]	2018	N/A	0–19 y	Canada	N/A	Abdomen	FAST and MSCT can detect injuries that may not be clinically apparent	Review
Rothrock, SG et al. [23]	2020	N/A	0–19 y	USA	N/A	Abdomen	A protocol for the management of children with abdominal trauma	Review
Stylianos S et al. [24]	2002	312	0/19 y	USA	1995–1997	Spleen and liver	The implementation of treatment guidelines based on injury severity	Prospective study
Djordjevic I et al. [25]	2021	76	0–18 y	Serbia	2016–2022	Abdomen	It is difficult to predict the further course of developing complications, but complications are more common in high-grade injuries	Prospective study
Costa G et al. [26]	2010	25,875	Children and adults	Italy	2005–2006	Abdomen	A high Injury Severity Score (ISS) is a predictor of mortality	Comparative study
Beuran M et al. [27]	2012	N/A	Children and adults	Romania	N/A	spleen	Definition of criteria for nonoperative management	Review
Cirocchi R et al. [28]	2013	16,940	Children and adults	Italy	2000–2011	spleen	Preserve the spleen whenever possible	Systematic review
Nouira F et al. [29]	2012	51	0–19 y	Tunisia	1996–2009	liver	The selection of patients for nonoperative treatment according to a CT scan and clinical stability	Retrospective review
Renz BM et al. [30]	1995	254	Children and adults	USA	1993–1994	All organs	Severe injuries are followed by a higher percentage of complications	Prospective study
Gaines BA et al. [31]	2010	N/A	Children and adults	USA	2003–2008	Abdomen and chest	Laparoscopy has become an important adjunct in the evaluation of both blunt and penetrating intra-abdominal trauma	Review
Cotton BA et al. [32]	2004	N/A	0–19 y	USA	N/A	Abdomen	Penetrating traumatic injuries have a significantly higher mortality rate than blunt injury	Review
Lankster MA et al. [33]	2005	N/A	0–19 y	USA	N/A	All organs	Definition of Pediatric Advanced Life Support Guidelines	Review
Deakin CD et al. [34]	1994	15,354	0–19 y	UK	N/A	All organs	Further studies are needed to establish optimum volume replacement in trauma patients with hypovolemic shock.	Review
García A et al. [35]	2021	16,593	Children and adults	Columbia	2002–2014	All organs	During the surgical procedure, immediate bleeding control must be achieved	Review
Samuels JM et al. [36]	2017	N/A	Children and adults	Romania	N/A	All organs	Personalized medicine will include changes in trauma care as more studies that assess coagulation, inflammation, and shock	Review
Polites SF et al. [37]	2017	2989	0–18 y	USA	2010–2014	Abdomen	Further studies are needed	Retrospective study
Leibner E et al. [38]	2020	N/A	Children and adults	USA	N/A	All organs	Damage control surgery improves survival	review
Tyburski JG et al. [39]	2001	470	Children and adults	USA	1998–2000	Abdomen	Rapid control of bleeding and hypothermia improves survival	Retrospective study
Kincaid EH et al. [40]	2001	515	0–12 y	USA	1998–2000	All organs	The risk of mortality increases precipitously in children with a base deficit of less than −8 mEq/L	Retrospective study
Fu Y et al. [41]	2023	879	Children and adults	USA	2015–2020	Liver	Gunshot liver injury is associated with a high incidence of liver-related complications	Retrospective study
Bieler D et al. [42]	2021	1123	Children and adults	Germany	2009–2018	Abdomen	In Germany, gunshot wounds have a low incidence	Retrospective study
Lasebikan OA et al. [43]	2019	55	Children and adults	Nigeria	2017–2018	Musculo-skeletal system	Effective control measures aimed at reducing the incidence of gunshot injuries will reduce the negative socioeconomic impact of these injuries	Prospective study
Omoke NI et al. [44]	2016	196	Children and adults	Nigeria	2005–2014	All organs	There is an increased risk of wound infection in gunshot injuries	Retrospective study

N/A, non-available; y, year.

## Data Availability

Not applicable.
